# Análisis de las cirugías cardíacas y mortalidad operatoria en el Instituto Nacional Cardiovascular durante el 2022

**DOI:** 10.47487/apcyccv.v4i2.287

**Published:** 2023-06-30

**Authors:** Gerber Polo-Gutierrez, Harod Anders Silva-Tejada, Franklin Willy Martinez-Ninanqui, Victor Robles-Velarde, Josías Ríos-Ortega

**Affiliations:** 1 Instituto Nacional Cardiovascular. Lima, Perú Instituto Nacional Cardiovascular Lima Perú

**Keywords:** Procedimientos Quirúrgicos Cardíacos, Evolución Clínica, Perú, Cardiac Surgical Procedures, Clinical Evolution, Peru

## Abstract

**Objetivo:**

. Describir las cirugías cardíacas, su abordaje y determinar la mortalidad operatoria según el tipo de cirugía y las principales complicaciones registradas hasta los 30 días del posoperatorio realizadas en el Instituto Nacional Cardiovascular de Perú.

**Materiales y métodos.:**

Se llevó a cabo un estudio descriptivo en todos los pacientes mayores de 18 años que fueron sometidos a cirugía cardiovascular en el Instituto Nacional Cardiovascular «Carlos Alberto Peschiera Carrillo».

**Resultados:**

. Durante el año 2022 se realizaron 503 cirugías cardíacas. De los pacientes intervenidos, el 63,6% (320) fueron varones. La cirugía valvular aislada, principalmente el reemplazo de la válvula aórtica o mitral, fue el procedimiento quirúrgico más frecuente, con 136 cirugías (27,0%). Le siguió la cirugía de revascularización de miocardio con 110 procedimientos (21,9%). A lo largo del año se registraron 23 fallecimientos, lo que resultó en una mortalidad general del 4,5%. La mortalidad en cirugías electivas fue del 2,8%, mientras que en cirugías de emergencia fue del 14,3%. La complicación más común fue la fibrilación auricular paroxística (14,0%), seguida de la infección del sitio quirúrgico con 52 casos (10,3%).

**Conclusiones:**

. La cirugía valvular, ya sea aislada o en combinación con otros procedimientos, fue realizada con más frecuencia. La tasa de mortalidad obtenida se considera aceptable para un centro de referencia.

## Abstract (en)

**Objective.:**

To describe cardiac surgeries, their approaches, and determine operative mortality according to the type of surgery and the main complications recorded within 30 days postoperatively, performed at the National Cardiovascular Institute of Peru.

**Materials and methods.:**

A descriptive study was conducted on all patients over 18 years of age who underwent cardiovascular surgery at the National Cardiovascular Institute “Carlos Alberto Peschiera Carrillo”.

**Results.:**

During the year 2022, a total of 503 cardiac surgeries were performed. Of the patients undergoing surgery, 63.6% (320) were males. Isolated valvular surgery, primarily aortic or mitral valve replacement, was the most frequent surgical procedure, with 136 surgeries (27.0%). This was followed by myocardial revascularization surgery with 110 procedures (21.9%). Throughout the year, there were 23 deaths, resulting in an overall mortality rate of 4.5%. The mortality rate for elective surgeries was 2.8%, while for emergency surgeries, it was 14.3%. The most common complication was paroxysmal atrial fibrillation (14.0%), followed by surgical site infection with 52 cases (10.3%).

**Conclusions.:**

Valvular surgery, whether isolated or combined with other procedures, was the most frequently performed. The obtained mortality rate is considered acceptable for a reference center.

## Introducción

Las enfermedades cardiovasculares (ECV) representan la principal causa de muerte a nivel mundial, con aproximadamente 17,5 millones de muertes cada año, de las cuales el 80% ocurren en países de ingresos bajos y medios ^1^. A pesar de su alta prevalencia en estas regiones, existe una escasez de datos precisos y de calidad, lo que posiblemente subestime la verdadera magnitud del problema a nivel mundial. La cirugía cardíaca ha sido reconocida recientemente como parte integral de los sistemas de salud nacionales; sin embargo, la Iniciativa Global de Cirugía Cardíaca indica que cerca de 6 mil millones de personas en todo el mundo no tienen acceso a una atención quirúrgica cardíaca segura cuando la necesitan ^2^.

La pandemia de la COVID-19 ha exacerbado aun más las desigualdades en salud a nivel global. Por ejemplo, en África, se estima que existe solo 1 cirujano cardiotorácico por cada 4 millones de habitantes ^3^. Esta situación resalta la necesidad urgente de incluir la cirugía cardíaca en los planes quirúrgicos de cada país. Para garantizar el acceso a la atención quirúrgica cardíaca para aquellos que no la tienen, es crucial realizar un mapeo adecuado del estado de la cirugía cardíaca a nivel nacional.

En Perú, la cirugía cardíaca se concentra principalmente en Lima, especialmente en los hospitales del seguro social, debido a la escasez de recursos en los hospitales públicos ^4^^,^^5^. El Instituto Nacional Cardiovascular de Perú es el principal centro de referencia en el país para el tratamiento de enfermedades cardiovasculares. Sin embargo, desde su creación en la década de 1990, no se ha publicado ningún trabajo que muestre los resultados del tratamiento quirúrgico de estas patologías. Por lo tanto, el objetivo del presente estudio es describir las principales cirugías cardíacas, su abordaje y determinar la mortalidad operatoria según el tipo de cirugía y las principales complicaciones registradas hasta los 30 días del posoperatorio realizadas en el Instituto Nacional Cardiovascular de Perú.

## Materiales y métodos

### Diseño y población de estudio

Se realizó un estudio descriptivo a partir de los registros de cirugías del Servicio de Cirugía Cardiovascular del Instituto Nacional Cardiovascular «Carlos Alberto Peschiera Carrillo» y del programa de historias clínicas de EsSalud (SGSS). El instituto es un centro especializado de referencia nacional de la seguridad social en Perú (EsSalud) para el tratamiento de enfermedades cardiovasculares de alta complejidad y que está ubicado en Lima, capital del Perú.

La población de estudio incluyó a todos los pacientes mayores de 18 años que fueron sometidos a cirugía cardiovascular durante el período del 1 de enero al 31 de diciembre de 2022. Se incluyeron todos los pacientes operados durante este período, sin exclusiones.

### Variables

Se incluyeron las siguientes variables de interés:

Tipo de cirugía. Se analizaron los siguientes subtipos de cirugías: cirugía valvular, que involucra uno o más procedimientos en las válvulas cardíacas; cirugía coronaria, procedimiento de bypass coronario aislado; cirugía coronaria con injertos multiarteriales, que implica dos o más anastomosis distales con conductos arteriales (arteria mamaria más arteria radial o doble arteria mamaria); cirugía valvular combinada, procedimientos que combinan cirugía valvular y coronaria; cirugía de aorta, que abarca diversas enfermedades como disección, úlcera penetrante, hematoma intramural, aneurisma y pseudoaneurisma; y otros procedimientos misceláneos con circulación extracorpórea (CEC) como cirugía de tumores cardíacos, miocardiopatía hipertrófica, extracción de cables de marcapasos infectados y tromboendarterectomía pulmonar.

Mortalidad operatoria. Definida como todas las muertes, independientemente de la causa, que ocurran durante la hospitalización en la que se realizó la operación, incluso después de 30 días.

Complicaciones posoperatorias. Ventilación mecánica prolongada, definida como intubación durante más de 48 horas en el período postquirúrgico; accidente cerebrovascular, confirmado por sospecha clínica y tomografía cerebral; sangrado excesivo, que requirió reintervención quirúrgica para exploración y revisión de hemostasia; reintervención cardíaca, que implicó una segunda operación con circulación extracorpórea para corregir complicaciones quirúrgicas dentro de los primeros 30 días después de la cirugía; infarto de miocardio, definido según la cuarta definición universal de infarto de miocardio; mediastinitis, infección profunda del sitio quirúrgico que requirió reintervención quirúrgica para limpieza.

### Análisis estadístico

Los datos fueron procesados utilizando el programa MS Excel y el programa estadístico Stata 15 (Stata Corporation, College Station, Texas, USA). Las variables categóricas se presentan como frecuencias absolutas y porcentajes, mientras que las variables cuantitativas se presentan como mediana y rango intercuartil debido a su distribución no normal.

### Aspectos éticos

Se respetaron los principios éticos de la Declaración de Helsinki, y todos los datos de los pacientes son anónimos.

## Resultados

En el año 2022 se realizaron un total de 503 cirugías cardíacas, de las cuales 320 (63,6%) correspondieron a pacientes varones. Del total de pacientes operados, 106 (21,0%) tenían menos de 50 años; 75 (14,9%) tenían entre 50 y 59 años; 165 (32,8%) tenían entre 60 y 69 años; 140 (27,8%) tenían entre 70 y 79 años, y 17 (3,3%) tenían más de 80 años.

La **Tabla 1** muestra el volumen de cirugías realizado durante cada mes del año 2022. Se observa que los primeros meses del año presentaron un menor número de cirugías debido a la reactivación progresiva de las actividades asistenciales después de la cuarentena estricta durante la pandemia de la COVID-19. La cirugía valvular aislada (principalmente reemplazo de válvula aórtica o mitral) fue el procedimiento quirúrgico más frecuente, con un total de 136 cirugías (27,0%). Le siguió la cirugía de revascularización de miocardio (*bypass* coronario) con 110 procedimientos (21,9%). Es importante destacar que se realizaron 12 trasplantes de corazón durante todo el año (2,4%).

**Tabla 1:** Tipos de cirugías realizadas en el Instituto Nacional Cardiovascular durante el 2022

Tipo de cirugía	Mes												Total	%
	Ene	Feb	Mar	Abr	May	Jun	Jul	Ago	Sep	Oct	Nov	Dic		
Cardiopatías congénitas	0	2	6	3	5	3	1	1	3	1	0	1	26	5,2
Combinada	6	6	3	2	3	8	1	4	5	3	1	1	43	8,5
Coronaria	8	8	7	7	8	6	5	9	11	14	10	17	110	21,9
De la aorta con CEC	3	2	8	5	5	4	3	3	4	3	7	4	51	10,1
Multivalvular	6	3	4	9	8	9	11	6	3	7	7	7	80	15,9
Valvular aislada	6	9	8	7	13	15	18	15	13	10	13	9	136	27,0
Reparación de complicaciones mecánicas posinfarto	0	0	0	3	2	2	1	2	0	1	1	2	14	2,8
Otras cirugías con CEC	2	2	1	3	5	1	2	2	2	1	3	0	24	4,8
Trasplante cardiaco	1	0	1	1	0	2	1	0	3	2	1	0	12	2,4
ECMO / Asistencia ventricular	0	0	0	0	0	0	1	0	5	1	0	0	7	1,4

CEC: circulación extracorpórea; ECMO: circulación por membrana extracorpórea.

En la Tabla 2 se detalla que el reemplazo de válvula aórtica fue la cirugía valvular más común (112 casos, 51,9% del total de cirugías valvulares y 22,3% del total de cirugías cardíacas realizadas en el año). La cirugía coronaria con el uso de dos injertos arteriales (arteria mamaria izquierda + arteria radial y arteria mamaria bilateral) se realizó en 30 pacientes (27,2% del total de cirugías coronarias).

**Tabla 2:** Tipos de cirugía realizados según el procedimiento en el Instituto Nacional Cardiovascular durante el 2022

Tipo de cirugía	n	%
Cirugía valvular	216	100
Cirugía valvular aislada	136	63,0
Reemplazo valvular aórtico	112	51,9
Reemplazo valvular mitral	10	4,6
Reemplazo valvular pulmonar	3	1,4
Reemplazo valvular tricúspide	2	0,9
Reparación valvular tricúspide	5	2,3
Reparación valvular aórtica	2	0,9
Reparación valvular mitral	2	0,9
Cirugía multivalvular	80	37,0
Cirugía de tres valvular	17	7,9
Reemplazo de una válvula + una reparación	42	19,4
Reemplazo de dos válvulas	17	7,9
Reparación de dos válvulas	4	1,9
Cirugía coronaria	110	100
Arteria mamaria + vena safena	78	70,9
Arteria mamaria + arteria radial	20	18,1
Arteria mamaria bilateral	10	9,1
Sólo vena safena	2	1,8
Cirugía combinada	43	100
Reemplazo valvular aórtico + revascularización	23	53,0
Reemplazo valvular mitral + revascularización	9	21,1
Doble reemplazo valvular + revascularización	8	19,0
Reparación valvular mitral + revascularización	3	7,0

En la Tabla 3 se describen la cantidad y el tipo de cirugías mínimamente invasivas realizadas (49 cirugías, 9,7% del total de cirugías). El reemplazo de válvula aórtica, ya sea por medio de minitoracotomía o miniesternotomía, fue la principal cirugía realizada por mínima invasión, con 23 procedimientos (46,9% del total de cirugías de mínimo acceso), seguida de la cirugía de válvula mitral y el cierre de comunicación interauricular (CIA).

**Tabla 3:** Cirugías por mínima-invasión en el Instituto Nacional Cardiovascular durante el 2022

Tipo de cirugía	n	%
Cirugías miniinvasivas	49	100
Reemplazo de válvula aórtica	23	46,9
Miniesternotomía superior	8	
Minitoracotomía anterior derecha	15	
Reemplazo de válvula aórtica + cirugía de aorta ascendente por miniesternotomia superior	02	4,1
Cirugía de válvula mitral por minitoracotomía	16	32,7
Reemplazo de válvula mitral	2	
Reemplazo de válvula mitral + reparación de tricúspide	8	
Reparación de válvula mitral	1	
Reparación de válvula mitral + reparación de tricúspide	4	
Reparación de válvula mitral + reparación de tricúspide + cierre de CIA	1	
Cierre de CIA por minitoracotomía	7	14,3
CIA osteum secundum	1	
CIA osteum secundum + reparación de tricúspide	4	
CIA seno venoso	2	
Revascularización de miocardio por minitoracotomía	1	2,0

CIA: comunicación interauricular.

Durante todo el año se registraron 23 fallecimientos, lo que representa una mortalidad general del 4,5%. Sin embargo, al calcular la mortalidad en cirugías electivas, esta fue del 2,8%, mientras que en cirugías de emergencia fue del 14,3%. La cirugía valvular aislada (reemplazo de válvula aórtica o cirugía de válvula mitral), que fue el procedimiento más frecuente, presentó una mortalidad del 0,8% en casos electivos. La cirugía coronaria aislada tuvo una mortalidad del 1,1% en pacientes estables, pero del 40,0% en casos de emergencia. La Tabla 4 muestra la mortalidad según los tipos de cirugías y la condición del paciente.

**Tabla 4:** Mortalidad general, según momento quirúrgico y tipo de cirugía en el Instituto Nacional Cardiovascular durante el 2022

Tipo de cirugía	Electiva			Emergencia		Total de casos (electiva y emergencia)	Total de muertes n (% sobre el total de del subtipo de cirugia)
	Total n	Mortalidad n (%)		Total n	Mortalidad n (%)		
Reparación de cardiopatías congénitas	26	0		0	0	26	0
Cirugía combinada	38	3 (7,9)		5	0	43	3 (6,9)
Cirugía coronaria	95	1 (1,1)		15	6 (40,0)	110	7 (6,3)
Cirugía de aorta con CEC	43	3 (7,0)		8	2 (25,0)	51	5 (9,8)
Cirugía multivalvular	76	3 (3,9)		4	0	80	3 (3,7)
Cirugía valvular aislada	127	1 (0,8)		9	1 (11,0)	136	2 (1,5)
Complicaciones mecánicas posinfarto	3	0		11	1 (9,0)	14	1 (7,1)
Otras con CEC	18	1 (5,5)		6	0	24	1 (4,1)
Trasplante cardiaco	0	0		12	1 (8,3)	12	1 (8,3)
ECMO / Asistencia ventricular	0	0		7	0	7	0
Total	426	12 (2,8)		77	11 (14,3)	503	23 (4,5)

CEC: circulación extracorpórea; ECMO: circulación por membrana extracorpórea.

La principal complicación registrada fue la fibrilación auricular paroxística, con una frecuencia del 14,0% en los primeros 30 días del postoperatorio. La infección del sitio quirúrgico fue la segunda complicación más frecuente, con 52 casos (10,3%). El sangrado posoperatorio excesivo que requirió reintervención para revisión de hemostasia se presentó en el 8,1% de los casos. Encontramos un total de 10 pacientes con accidente cerebrovascular confirmado por imágenes de tomografía cerebral y síntomas clínicos sugestivos (2,0% del total). La Tabla 5 muestra las principales complicaciones registradas.

**Tabla 5:** Complicaciones posoperatorias

Complicación	n (%)
Ventilación mecánica prolongada	20 (4,0)
Stroke	10 (2,0)
Sangrado posoperatorio excesivo	41 (8,1)
Reintervención cardiaca	4 (0,8)
Marcapaso permanente	10 (2,0)
Fibrilación auricular paroxística	70 (14,0)
Infarto de miocardio perioperatorio	6 (1,2)
Mediastinitis	3 (0,6)
Infección de sitio operatorio	52 (10,3)

En la Tabla 6 se muestra la estancia en la unidad de cuidados intensivos (UCI) y la estancia hospitalaria total. La cirugía de trasplante cardíaco tuvo la mayor estancia tanto en UCI (mediana de 11 días, rango: 10-14) como en el hospital (mediana de 25 días, rango: 22-29). La cirugía de revascularización de miocardio tuvo una mediana de estancia en UCI de 2,5 días (rango: 2-4) y en el hospital de 8 días (rango: 7-12). La cirugía valvular aislada (cambio de válvula aórtica o cirugía de válvula mitral) tuvo una estancia en UCI de 3 días (rango: 2-5) y en el hospital de 10 días (rango: 8-16).

**Tabla 6:** Estancia en la unidad de cuidados intensivos y estancia hospitalaria según tipo de cirugía

Tipo de cirugía	Estancia en UCI (días)*	Estancia hospitalaria (días)*
Cardiopatías congénitas	6,5 (2-8)	11 (7-23)
Combinada	5 (2-12)	9 (8-28)
Revascularización miocárdica	2,5 (2-4)	8 (7-12)
Aorta con CEC	4 (2-5)	11 (8-25)
Multivalvular	3 (3-5)	10 (8-17)
Valvular aislada	3 (2-5)	10 (8-16)
Reparación de complicaciones mecánicas posinfarto	7 (5-8)	16 (14-20)
Otras cirugías con CEC	3 (2-7)	8 (7-15)
Trasplante cardiaco	11 (10-14)	25 (22-29)

CEC: circulación extracorpórea; UCI: unidad de cuidados intensivos.

* Datos expresados en mediana y rango intercuartil.

## Discusión

Durante el año 2022 se llevaron a cabo un total de 503 cirugías cardíacas. La cirugía valvular aislada, principalmente el reemplazo de la válvula aórtica o mitral, fue el procedimiento quirúrgico más común, con un total de 136 cirugías (27,0%), seguido de la cirugía de revascularización de miocardio con 110 procedimientos (21,9%). A lo largo del año se registraron 23 fallecimientos, lo que resultó en una mortalidad general del 4,5%. En cirugías electivas la mortalidad fue del 2,8%, mientras que en cirugías de emergencia fue del 14,3%.

En este estudio hemos informado por primera vez la actividad quirúrgica cardiaca de forma global en nuestro hospital, lo cual complementa los escasos estudios publicados de otros centros a nivel nacional y latinoamericano ^6^^-^^8^. En cuanto al análisis mensual, observamos una disminución en la cantidad de cirugías durante los primeros dos meses en comparación con el resto del año 2022. Esto indica que nuestro centro no fue ajeno al impacto de la pandemia, siendo enero y febrero los meses más cercanos a la etapa pospandemia. Un fenómeno similar se observa en el registro español de cirugía cardiovascular del 2020, el cual muestra una disminución de casi el 20% en la actividad en este contexto ^9^.

Destacamos en nuestros resultados que las cirugías valvulares son las más frecuentes, al igual que en México, donde se reportan resultados similares a nuestra realidad, con la cirugía cardiaca valvular aislada como la más común, seguida de la cirugía coronaria ^7^. Esto difiere de los registros de países desarrollados como Estados Unidos, donde predomina la cirugía coronaria (70%) ^10^. En Argentina, Lowenstein *et al*. informan que la cirugía coronaria es la más frecuente, por encima de las cirugías valvulares ^6^. Gomez *et al*. en Brasil reportan que la cirugía de revascularización miocárdica es la más realizada, representando el 48,8%, seguida de la cirugía valvular (23,3%) ^11^.

Dentro de las cirugías valvulares el reemplazo valvular aórtico aislado se mantiene como el procedimiento más frecuente, un hallazgo similar a los resultados de Lowenstein *et al*. y al registro del año 2022 de la STS (The Society of Thoracic Surgeons) ^6^^,^^10^.

Respecto a los injertos utilizados en la cirugía coronaria, la asociación de arteria mamaria más vena safena fue lo más utilizado, representando el 70,9%. En segundo lugar, los injertos multiarteriales (mamaria más radial y doble mamaria) representaron el 27,3%, lo cual es mayor en comparación con los resultados del registro del año 2022 de la STS, que informan el uso de estos injertos en el 14,3% de las cirugías coronarias ^10^. En nuestros resultados se observa que el injerto radial se utilizó como segundo conducto arterial en un 18,1%, siendo también una preferencia similar al registro de la STS ^10^. El mayor uso de este injerto en nuestra institución, durante el 2022, se basa en los resultados de los estudios liderados por Gaudino y por Quereshi, en los cuales el uso de injertos de arteria radial para la revascularización coronaria resultó en una tasa más baja de eventos cardíacos adversos y una mayor permeabilidad a los 5 años de seguimiento en comparación con el injerto de safena ^12^^-^^14^.

Lowenstein *et al*. encontraron que la cirugía combinada más frecuente es el reemplazo aórtico más revascularización coronaria en un 79,5% ^6^. Sin embargo, en nuestro centro este subtipo de intervención resultó en un 53%, hallazgo similar a los resultados de los últimos 3 años del registro de la STS ^(^^15^^,^^16^^).^

En cuanto a los datos de cirugía de emergencia, la cirugía coronaria fue la más frecuente, con un 19,5% (15 de 77 casos), seguida del trasplante cardíaco con un 15,6% (12 de 77 casos). Dado que somos un centro de referencia nacional, el trasplante cardíaco abarca un porcentaje considerable de los casos de emergencia. Los datos de Mitrev y Anguseva, también encuentran que la cirugía coronaria fue la intervención quirúrgica cardiaca más frecuente en el contexto de emergencia, pero con un mayor porcentaje (45,47%), probablemente este mayor valor se deba a que su estudio aborda mayor cantidad de pacientes y más años de seguimiento ^17^.

La cirugía cardíaca actualmente busca enfoques menos invasivos y una recuperación más rápida. En nuestro estudio se utilizó la técnica mínimamente invasiva en un 9,7% (49 de 503) de todas las cirugías cardiacas del año 2022, un porcentaje menor en comparación con el 38,7% registrado por la STS en el año 2021 ^(^^15^^).^ La menor adopción de este abordaje en nuestro estudio se debe a la necesidad de contar con personal capacitado y disponibilidad de recursos adicionales.

Respecto a las complicaciones posoperatorias, la fibrilación auricular fue la más frecuente en nuestros resultados, con una incidencia del 14% en el período de 30 días después de la cirugía. Esta complicación también es la más común en los resultados del registro de la STS, presentándose en el 26% de los pacientes sometidos a cirugía de revascularización coronaria y en el 27% de los pacientes sometidos a reemplazo valvular aislado ^18^. Sin embargo, estos datos difieren de los resultados de Pahwa S. *et al*., donde se evidenció que la complicación posoperatoria más frecuente fue el sangrado posoperatorio con un 47,3%, seguido de la fibrilación auricular posoperatoria con un 32% ^19^.

La tasa de mortalidad global informada en nuestra institución fue mayor a la reportada por Salamanca *et al*. en un estudio descriptivo realizado en un hospital general de Perú (4,5% vs. 0%) ^8^, teniendo en cuenta que Salamanca reporta un número de casos mucho menor al nuestro y una complejidad menor de los tipos de intervenciones cardiacas. Sin embargo, nuestra mortalidad es menor que la reportada por Rodríguez-Hernandez (4,5% vs. 9,2%), que la reportada por el registro brasileño (4,5% vs 6,4%) y de la misma forma que la indicada por el registro español (4,5% vs 5,5%), en un contexto de centros de referencia nacionales y donde la complejidad quirúrgica se asemeja más al de nuestra institución ^7^^,^^9^^,^^11^. 

La tasa de mortalidad de los 110 pacientes que se sometieron a cirugía coronaria fue del 6,3 %, la cual fue más alta que la tasa de mortalidad internacional (2,7 a 4,9%) ^7^^,^^9^^,^^10^. Pero si tenemos en cuenta solo las cirugías coronarias electivas, nuestra mortalidad (1,1%) estaría por debajo de las mencionadas cifras internacionales. Sin embargo, estos registros internacionales no hacen una diferencia entre cirugía de emergencia o electiva. 

Si tomamos atención en nuestra casuística coronaria de emergencia observamos que nuestra mortalidad (40%) es considerablemente superior a lo que se reporta en un centro estadounidense por Schumer et al., la cual es de 8,7% ^20^; pero se acerca a la reportada por Oliveira et al., quienes reportan que en un hospital público del Sur de Brasil la mortalidad en pacientes sometidos a cirugía coronaria de emergencia fue del 36,4% ^21^. En este escenario, se requiere un análisis cuidadoso de los aspectos que sustentan estos datos, por lo que implica una oportunidad para realizar un estudio especifico en cirugía coronaria de los casos de nuestra institución para obtener datos importantes para mejorar los resultados para estos pacientes.

Ahora, respecto a la mortalidad operatoria para el reemplazo valvular aislado informamos que esta fue del 1,5%, cifra ligeramente menor al reporte de Kim *et al*., quienes indican una mortalidad para este subtipo de cirugía cardiaca del 2,3% ^10^. Otra ligera diferencia también ocurre en la cirugía combinada, ya que los resultados reportados por Siregar *et al*., indican que la cirugía combinada presentó una mortalidad del 5,3% ^22^, mientras que en nuestro resultado fue del 6,9%.

Este estudio presenta algunas limitaciones, ya que al ser retrospectivo depende de la información recopilada de las historias clínicas. Además, se llevó a cabo en una sola institución, lo que puede limitar la generalización de los hallazgos a otros entornos. También es importante tener en cuenta que este estudio solo informa sobre los procedimientos quirúrgicos realizados en el año 2022, por lo que puede que no represente las tendencias a lo largo del tiempo o en otros lugares. Debido a la falta de un registro a nivel nacional, siendo nuestro instituto un centro de referencia en el tratamiento de la patología cardiaca, la iniciativa de este estudio es crear una base de datos sobre la patología cardiaca y de la aorta que requieren intervención quirúrgica. A pesar de estas limitaciones es importante destacar que este es el primer informe a nivel nacional sobre los resultados de la cirugía cardíaca, lo cual resalta la importancia de nuestro estudio.

En conclusión, la cirugía valvular, ya sea aislada o en combinación con otros procedimientos, fue la más frecuentemente realizada en nuestra institución. Consideramos que la tasa de mortalidad y la frecuencia de complicaciones son aceptables y comparables con otros centros de referencia, pero para la cirugía coronaria se requiere un análisis cuidadoso, por lo que implica una oportunidad para realizar un estudio especifico en este suptipo de cirugía cardiaca, para obtener datos importantes para mejorar los resultados para estos pacientes.
